# A Suspension-Trapping Protocol for Bottom-Up Proteomics Sample Preparation

**DOI:** 10.21769/BioProtoc.5681

**Published:** 2026-05-05

**Authors:** Joseph Schrader, Dennis Province, Nicholas A. DaSilva, Chang Liu

**Affiliations:** 1 Department of Biomedical and Pharmaceutical Sciences, College of Pharmacy, University of Rhode Island, Kingston, RI, USA; 2 IDeA National Resource for Quantitative Proteomics, University of Arkansas for Medical Sciences, Little Rock, AR, USA; 3 Proteomics Core Facility, Department of Molecular Biology, Cell Biology, and Biochemistry, Brown University, Providence, RI, USA; 4 Proteomics Facility, College of Pharmacy, University of Rhode Island, Kingston, RI, USA

**Keywords:** Mass spectrometry, LC-MS, Proteomics, Sample preparation, S-trap

## Abstract

Bottom-up proteomics workflows encompass several key stages, including sample preparation, data acquisition, and data analysis. Of these, sample preparation is the initial and critical stage, as it significantly influences the depth, reproducibility, and reliability of subsequent mass spectrometry–based analyses. While several main digestion strategies exist, including in-gel, in-solution, and filter-aided methods, each presents distinct trade-offs in terms of throughput, contamination removal, and applicability to complex biological matrices. The Suspension Trapping (S-Trap) method offers a compelling alternative by efficiently capturing and digesting proteins while removing interferents like sodium dodecyl sulfate (SDS), which can compromise downstream LC–MS/MS performance. This protocol details a S-Trap workflow optimized for biofluid proteomics, specifically plasma, serum, and cerebrospinal fluid (CSF). We describe two complementary formats: a manual tube-based procedure for individual or small-batch samples and a 96-well-plate-based system enabling high-throughput processing. The protocol integrates optional high-abundance protein depletion to enhance coverage of low-abundance analytes and includes steps for reduction, alkylation, digestion, and peptide elution for low total protein content samples, such as plasma, serum, and cerebrospinal fluid. By providing a detailed protocol, this work aims to improve the consistency and accessibility of S-Trap-based sample preparation, facilitating robust and reproducible discoveries in bottom-up proteomics.

Key features

• Plasma/serum/cerebrospinal fluid sample preparation for bottom-up proteomics.

• Lab Suspension Trapping (S-Trap)-based digestion for efficient detergent removal and high peptide recovery.

• Optimized for challenging samples (e.g., CSF, plasma) with low protein concentration or high lipid content.

• Includes both single-tube and high-throughput 96-well plate formats for flexible experimental design.

## Graphical overview


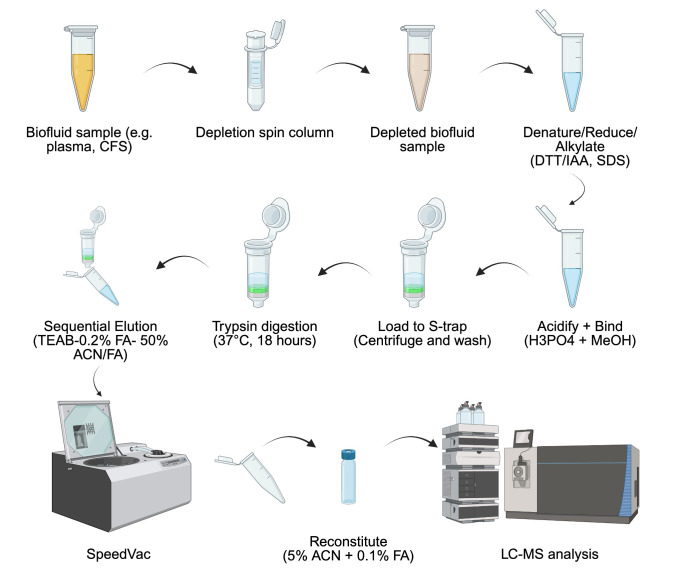


**Biofluid sample Suspension Trapping (S-Trap) protein extraction workflow for bottom-up proteomics analysis**

## Background

In the field of bottom-up proteomics, protein digestion strategies are primarily categorized into in-gel, in-solution, and on-filter digestion, each offering unique strengths and limitations already summarized [1,2]. Additionally, comparative studies that aimed to address the advantages and disadvantages of different approaches have also been reported [2]. In-solution digestion is one of the most commonly used methods for tissue and cell samples [3]. It involves denaturing, reducing, alkylating, and digesting proteins in the liquid phase, offering simplicity and operational efficiency [3]. Compared to in-gel digestion, this method minimizes sample loss and is less labor-intensive, making it particularly suitable for routine proteomic workflows [2]. However, challenges such as protein aggregation and incomplete solubilization can contribute to sample loss. In-gel digestion, by contrast, separates proteins based on size via SDS-PAGE before digestion [4]. This approach effectively reduces contaminants and allows for the fractionation of complex samples, enhancing mass spectrometry analysis for high-complexity proteomes [4]. Proteins are excised from the gel, destained, and digested directly within the gel pieces. While robust and reproducible, in-gel digestion is time-consuming and requires significant manual effort, which can limit throughput [2,4]. On-filter digestion, exemplified by filter-aided sample preparation (FASP), processes proteins retained on molecular-weight cutoff membranes, removing contaminants and recovering peptides through centrifugation [2,5]. FASP is widely used due to its robustness against detergents and gel-free nature [6]. However, the reliance on repetitive centrifugation, requiring over 10 cycles for a complete workflow, significantly increases processing time [2]. Recent adaptations, such as performing cell lysis, reduction, and alkylation outside the filter, have attempted to address this bottleneck, though limitations inherent to membrane-based approaches persist.

The Suspension Trapping (S-Trap) method, introduced by Zougman et al. in 2014, has gained significant attention in proteomics due to its efficiency, simplicity, and high protein recovery [7]. Its key features include a quartz fiber filter stack and a unique protein solubilization protocol using 5% SDS, 12% phosphoric acid, and 90% methanol [7]. This process creates protein particulates that are trapped on the filter, followed by in-filter digestion and peptide elution. The availability of commercial S-Trap filters and 96-well plates further streamlines workflows, improving reproducibility and reducing manual variability.

Sample preparation is a crucial yet challenging step in bottom-up proteomics, often hindered by time constraints, user variability, and labor-intensive processes [8]. Even within the same category of digestion protocols—whether in-gel, in-solution, or on-filter—variations in methods, chemicals, and reagents across proteomics facilities and research groups can significantly impact results [8]. Recognizing the importance of standardization, we previously established a robust chloroform/methanol protein extraction and in-solution trypsin digestion protocol [9]. Building on this foundation, we now expand our workflow to include the S-Trap method, providing a detailed protocol to support consistency and reproducibility. The peptides generated in this protocol are compatible with most liquid chromatography systems and data acquisition methods, including data-dependent acquisition (DDA) and data-independent acquisition (DIA). Moreover, this protocol includes both a tube format and a 96-well plate format, optimizing it for high-throughput proteomics analysis.

## Materials and reagents

**Biological materials**

1. Frozen rat cerebrospinal fluid sample

*Note: In our work, 31 samples were used. This protocol has also been successfully conducted using human cerebrospinal fluid and plasma samples.*

**Reagents**

1. Trizma base (Tris) (Sigma-Aldrich, catalog number: T1503-1KG)

2. Phosphoric acid (Honeywell, catalog number: 79606-500 mL)

3. Pierce^TM^ BCA Protein Assay kit with Dilution-Free^TM^ BSA protein standards (Thermo Fisher Scientific, catalog number: A55865).

4. High-Select^TM^ Top14 abundant protein depletion resin (Thermo Fisher Scientific, catalog number: A36366)

5. S-Trap^TM^ micro column (≤100 μg; PROTIFI, CO_2_-micro-10, CO_2_-micro-40, or CO_2_-micro-40, depending on numbers of samples processed)

6. Dithioreitol (DTT) (Sigma-Aldrich, catalog number: D9779-1G)

7. Iodoacetamide (IAA) (Sigma-Aldrich, catalog number: I1149-5G)

8. Trypsin w/ CaCl_2_ (TPCK-treated, 500 μg), 10 pack (SCIEX, catalog number: 4445250)

9. Milli-Q water

10. Formic acid (Sigma-Aldrich, catalog number: 695076-100ML)

11. Acetonitrile (ACN) CHROMASOLV LC-MS (Honeywell, catalog number: 34967-4X4L)

12. Sodium deoxycholate (DOC) (Sigma-Aldrich, catalog number: 30970-25G)

13. Ammonium bicarbonate (ABC) (Sigma-Aldrich, catalog number: A6141-25G)

14. Triethylammonium bicarbonate buffer (TEAB) (Sigma-Aldrich, catalog number: T7408-100 mL)

15. Dithiothreitol (DTT) solution, 1 M (ThermoFisher Scientific, catalog number: P2325)

16. PBS solution, pH 7.4 (1×, 10 mM) (Gibco^TM^, ThermoFisher Scientific, catalog number: 10010023)

*Note: Trypsin platinum, mass spectrometry grade (100 μg, Promega, catalog number: VA900) is recommended for samples such as cerebrospinal fluid. For tissue or cell lysate samples, suitable alternatives include trypsin platinum, mass spectrometry grade (100 μg, Promega, catalog number: V5280) or sequencing-grade modified trypsin (lyophilized, 5 × 20 μg, Promega, catalog number: V5111)*

**Solutions**

1. Lysis buffer (see Recipes)

2. 50 mM ammonium bicarbonate (pH ~8) containing 3% (w/v) DOC (see Recipes)

3. Aqueous buffer (see Recipes)

4. S-trap binding buffer, pH 7.1 (see Recipes)

5. Buffer A (see Recipes)

6. Buffer B (see Recipes)

**Recipes**

**1. Lysis buffer**

**Table:** 

Reagent	Final concentration
SDS	2%
Tris (pH 8.5)	100 mM

*Note: The composition of the lysis buffer may vary in SDS concentration, typically 2% or 10%. SDS functions primarily to denature proteins, prevent aggregation, and maintain protein solubility. Higher concentrations (e.g., 10%) can be helpful for challenging samples such as skin tissue or membrane protein complexes; however, they also increase the complexity of downstream cleanup. In this protocol, we used 2% SDS, which was effective for cerebrospinal fluid (CSF) samples. A detailed recipe for high-concentration SDS and other buffer formulations is available upon request.*

**2. 50 mM ammonium bicarbonate (pH ~8) containing 3% (w/v) DOC**

**Table:** 

Reagent	Final concentration
Ammonium bicarbonate	50 mM
DOC	3%

**3. Aqueous buffer**

**Table:** 

Reagent	Final concentration
Acetonitrile	47.5%
Water	47.5%
Formic acid	5%

**4. S-trap binding buffer, pH 7.1**

**Table:** 

Reagent	Final concentration
Methanol	90%
TEAB	1 M

*Note: Rinse glassware with 100% methanol before preparing S-Trap binding buffer.*

**5. Buffer A**

**Table:** 

Reagent	Final concentration
Water (LC-MS grade)	99.4%
Acetonitrile	0.5%
Formic acid	0.1%

**6. Buffer B**

**Table:** 

Reagent	Final concentration
Acetonitrile	99.9%
Formic acid	0.1%

**Laboratory supplies**

1. Pipette tips (TipOne 10, 200, 1,000 μL) (USA Scientific, catalog numbers: 1111-3800, 1110-1800, 1111-2821)

2. Pipettes (ErgoOne single-channel pipette 2.5, 10, 200, 1,000 μL) (USA Scientific, catalog numbers: 7100-0125, 7100-0510, 7100-2200, 7110-1000)

3. Pour boat weigh dish 2-1/4”l ×1-3/4”w × 5/16”d, 20 mL cap (Wilkem Scientific, catalog number: 10177901)

4. Pipette tip gel loading 0.57 mm O.D. 200 μL round non-sterile (Wilkem Scientific, catalog number: LABB13790)

5. Microcentrifuge tube 0.5 mL non-sterile (Cell Treat, Wilkem Scientific, catalog number: 72316004)

6. Microcentrifuge tube 1.5 mL non-sterile (Cell Treat, Wilkem Scientific, catalog number: 229441)

7. Microcentrifuge tube 1.7 mL non-sterile (Cell Treat, Wilkem Scientific, catalog number: 229441)

8. S-trap^TM^ 96-well mini plate (PROTIFI, catalog number: P002-96MINI)

9. 96-well sample collection plate (Waters, catalog number: 186002842)

10. Sealing mat for 96-well plate, polypropylene, round well (Waters, catalog number: 186002483)

11. 96-well filter plate, 0.2 µm, 1 mL (AcroPrep Advance, Cytiva, catalog number: 8686)

*Note: Low protein-binding microcentrifuge tubes (e.g., SARSTEDT, catalog number: 72.706.600) are recommended to minimize peptide adsorption and improve recovery, especially for protein-limited samples such as cerebrospinal fluid.*

## Equipment

1. Eppendorf Vacufuge plus 5305 (Eppendorf AG, catalog number: 5305FQ525373)

2. Savant SpeedVac SPD210 vacuum concentrator (Thermo Scientific, catalog number: SPD210-P1)

3. Nanodrop One (ThermoFisher, catalog number: ND-ONE-W)

4. Eppendorf Thermomixer F1.5 (Eppendorf AG, model: 5384)

5. Grant-bio PMS-1000i (Grant Instrument Ltd., model: V.2GW.001)

6. Vortex MAXI MIX 1 (Thermo Scientific, Model: M16715)

7. Barnstead Thermolyne LABQUAKE Shaker Rotisserie (catalog number: 3625485)

8. Tecan (Resolvex) A200 Positive Pressure Manifold System

9. Thermo Fisher Orbitrap Ascend Tribrid Mass Spectrometer

## Software and datasets

1. Spectronaut^®^ (Version 18.0, Biognosys AG)

3. Rat UniProt FASTA database (UP000002494_10116_Rat_Reference_20240602.fasta) and Universal Contaminant Protein FASTA (created 06.01.22, used 06.03.25) were used for protein identification and to generate the library for DirectDIA analysis

## Procedure

**A. Preparation of chemicals and reagents (tube format)**

1. Prepare lysis buffer as described in Recipes.

*Note: For each sample, mix 125 μL of 10% SDS with 500 μL of 100 mM Tris.*

2. Prepare DTT working solution (160 mM): Dissolve 15.4 mg of DTT in 1 mL of Milli-Q water.

3. Prepare IAA solution: Add 36.98 mg of IAA (4 °C) to 1 mL of Milli-Q water.

4. Prepare phosphoric acid solution (12%): Mix 420 μL of 85% H_3_PO_4_ stock with 2,580 μL of Milli-Q water.

5. Prepare S-trap binding buffer as described in Recipes.

6. Prepare trypsin stock: Resuspend trypsin in 50 mM Tris, pH 8.5, at a concentration of 1 mg/mL.

7. Prepare elution buffer 1 (50 mM TEAB): Mix 100 mM TEAB with the same volume of Milli-Q water.

8. Prepare elution buffer 2 (0.2% formic acid).

9. Prepare elution buffer 3 (50% acetonitrile in water with 0.2% formic acid).

10. Prepare aqueous buffer as described in Recipes.

**B. Depletion of Top 14 high-abundance proteins**

**B1. Top 14 protein depletion (tube format)**

1. Equilibrate the depletion spin column to room temperature for at least 5 min.

2. Remove the screw cap from the column.

3. Add up to 80 μL of the CSF sample directly to the resin slurry in the column.

4. Securely cap the column and invert several times to homogenize the resin.

5. Incubate for 10 min at room temperature with gentle end-over-end mixing. Alternatively, vortex gently every 2–3 min. Incubation time can be extended to 20 or 30 min to increase depletion efficiency.

6. After incubation, remove the bottom closure by snapping it off.

7. Loosen the top cap and place the column into a 2 mL collection tube. Centrifuge at 1,000× *g* for 2 min.

8. Following centrifugation, discard the used column containing the resin and retain the filtrate, which is the sample depleted of high-abundance proteins.

9. Resuspend the sample in 10 mM PBS (pH 7.4, 1×) containing 0.02% sodium azide, pH 7.4. Proceed with downstream processing or store the sample at -20 °C for later use.

10. Transfer the collection tube to an Eppendorf Vacufuge. Set up 4 h and 30 °C on the V-AQ mode to dry the sample. Alternatively, the Centrivap step may be done at room temperature to avoid sample loss.

*Note: High-Select^TM^ Top14 Abundant Protein Depletion Resin has a different catalog number. A36369 and A36370: Mini spin columns with a 200 μL antibody resin bed, optimized to bind up to 600 μg of serum or plasma; A36371 and A36372: Midi spin columns with a 500 μL antibody resin bed, optimized to bind up to 6,000 μg of serum or plasma. After step B1.10, check if there is any liquid remaining in the collection tube. If so, extend the centrifugation time as needed. Protein concentration can be estimated using a NanoDrop following step B1.10. To optimize the current procedure, we avoid concentrating samples with heat, as elevated temperatures may cause artifacts and sample loss. Instead, turn off the temperature setting, use a chilled Centrivap, or employ a Labconco lyophilizer.*

**B2. Top 14 protein depletion (plate format)**

**Figure 1.**
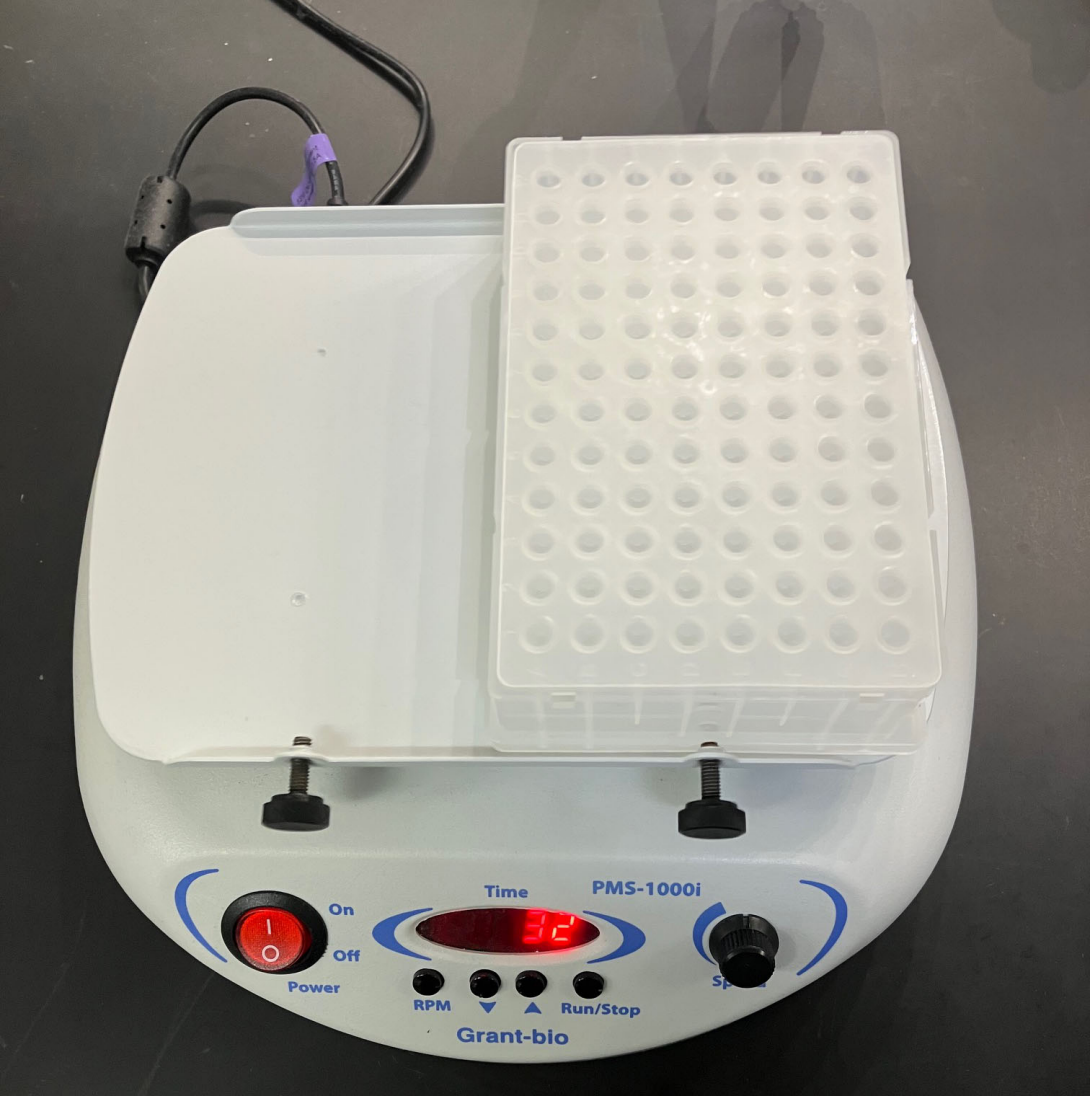
Placement of the 96-well collection plate on the plate rotator for mixing

1. Add 200 μL of Pierce High-Select Top 14 abundant protein depletion resin to a 0.8 mL 96-well storage plate.

2. Add 40 μL of CSF sample to each well.

3. Seal the plate with a round-top sealing mat.

4. Holding the sealing mat and storage plate firmly, vortex moderately for 30 s.

5. Ensure the mat is properly sealed and then carefully invert the plate several times to mix the solution thoroughly. Return the plate to the upright position and then vortex for 5 min (Figure 1).

6. Secure the plate on a plate rotator and rotate for 20 min.

7. Pulse centrifuge the plate for 10–15 s (with the mat still on) to push the resin to the bottom of the wells.

8. Remove the sealing mat.

9. Mix the contents using a multichannel pipette and transfer to an Acro 8686 96-well filter plate positioned on top of a 2 mL square-well collection plate.

10. Add 200 μL of PBS (10 mM, pH 7.4, 1×) to the storage wells, mix by pipetting, and transfer the remaining resin to the filter plate.

11. Centrifuge at 8,000× *g* for 5 min to push the liquid through the filter into the collection plate.

12. Place the collection plate on a SpeedVac concentrator to dry.

**C. Proteolytic digestion using S-trap**

**C1. Trypsin digestion (tube format)**

1. Resuspend samples in 175 μL of 1× lysis buffer in a collection tube (see Recipes).

*Note: A normalized amount of protein (e.g., 25 μg) could be combined with an equal volume of 2× lysis buffer to achieve a final concentration of 2% SDS in either 50 mM TEAB or 50 mM Tris. The total protein input is maintained within the recommended digestion capacity of the S-Trap columns (10–100 μg total protein), corresponding to an effective working concentration range of approximately 0.2–1 μg/μL for starting materials (e.g., plasma, cerebrospinal fluid, and plasma samples).*

2. Add 25 μL of 160 mM DTT into the collection tube for a final concentration of 20 mM. Mix by pipetting while adding (the 160 mM DTT working solution can be prepared from DTT powder or by dilution from a 1 M DTT stock solution).

3. Cover the collection tube and heat at 95 °C for 15 min on the thermomixer with shaking at 300 rpm (approximately 0.3× *g*, 3 mm orbit). For laboratories without a thermomixer, static incubation at 95 °C with gentle mixing of the tube every 5 min can help achieve even heating and prevent localized overheating.

*Note: Incubation at 70 °C for 30 min has proven effective. At sufficient concentrations, such as 10% SDS, denaturing proteins and larger proteins such as immunoglobulins are removed, leaving primarily lower-molecular-weight proteins, which can typically be denatured at 37 °C for 1 h or 55 °C for 45 min with shaking. For difficult-to-denature proteins or membrane-bound fractions, incubation at 70 °C for 30 min is recommended.*

4. Allow the sample to cool to room temperature (approximately 10 min).

5. Add 25 μL of 360 mM IAA for a final concentration of 40 mM and incubate in the dark for 30 min.

6. Add 16.1 μL of 12% phosphoric acid for a final concentration of 1.2% and mix by pipetting.

7. Add 600 μL of freshly made S-trap binding buffer (see Recipes) and mix by pipetting. The solution will turn cloudy as the protein forms a colloid.

8. Label and place the S-trap spin column (cap must be loosely placed on top) on a 2 mL microcentrifuge tube to collect the waste.

9. Add 250 μL of sample from the collection tube into the S-trap spin column.

10. Centrifuge at 4,000× *g* for 30 s to pass samples through the S-trap spin column.

11. Continue to add samples (200 μL at one time with centrifugation after each step) until completely loaded onto the S-trap spin column.

*Note: The maximum loading capacity of this S-Trap column is 100 μg in this study, as indicated in the reagents section. The choice of S-Trap column size is determined based on the protein amount after Top 14 protein depletion.*

12. Wash the S-trap spin column with S-trap binding buffer (300 μL each time, three times in total).

13. Transfer the S-trap spin column to a new clean 2 mL microcentrifuge tube.

14. Add 115 μL of 50 mM ABC to the S-trap spin column.

15. Add 10 μL of trypsin solution, cap the S-trap spin column, put it on the rack, and then put it into the incubator at 37 °C for 18 h.

**C2. Trypsin digestion (plate format)**

1. Resuspend samples in 50 μL of 1× lysis buffer.

2. Using a multichannel pipette, add 5 μL of 110 mM DTT for a final concentration of 10 mM (the 110 mM DTT working solution can be prepared from DTT powder or by dilution from a 1 M DTT stock solution). Mix by pipetting.

3. Cover the deep-well plate with a sealing mat and heat at 95 °C for 15 min with shaking/mixing.

*Note: Denaturation, reduction, alkylation, and acidification can be done in 1.5 mL low protein-binding tubes and then transferred to a S-Trap plate.*

4. Allow to cool to room temperature (~10 min) (alternatively, centrifuge for 2 min).

5. Add 5 μL of 240 mM IAA (prepared fresh immediately before use; light-sensitive) for a final concentration of 20 mM and incubate in the dark for 30 min.

6. Add 5 μL of 32.5% phosphoric acid for a final concentration of 2.5%. Mix by pipetting.

7. Add 390 μL of freshly prepared S-Trap binding buffer and mix by pipetting. The solution will turn cloudy as proteins form a colloid.

8. Label the S-Trap plate and place it on a square-well collection plate to collect waste.

9. Add 250 μL of sample to the S-Trap plate at a time (repeat until the entire sample is transferred). Using the Tecan A200 Positive Pressure Manifold, apply increasing pressure (i.e., Flash) until all liquid has passed through. Alternatively, centrifuge the plate at 800× *g* for 2–5 min to pass the liquid through the membrane.

10. Wash 4 times with 300 μL of S-Trap binding buffer, applying positive pressure as in the previous step.

11. Place the S-Trap plate on top of a clean 2 mL deep-well collection plate.

12. Add 150 μL of 50 mM ABC containing sufficient trypsin (1:50 minimum enzyme to protein ratio; 1:100 may lead to incomplete digestion and large peptide fragments) using a single or multichannel pipette. Cover and place in a suitable ziplock bag with a damp Kimwipe to provide humidity and prevent evaporation of trypsin solution. Alternatively, cover the plate with a clear plastic lid and incubate, placing a pan of water in the incubator to prevent the plate from drying out. Incubate for 18 h at 37 °C.

**D. Peptide elution from S-trap**

**D1. Elution (tube format)**

1. Transfer the rack to room temperature. After removing the S-trap column cap on top of the S-trap spin column, add 40 μL of elution buffer 1 to the S-trap and then centrifuge at 4,000× *g* for 1 min.

2. Add 40 μL of elution buffer 2 to the S-trap and then centrifuge at 4,000× *g* for 1 min.

3. Add 40 μL of elution buffer 3 to the S-trap and then centrifuge at 4,000× *g* for 1 min). This elution assists in the recovery of hydrophobic peptides.

4. Pool eluted peptides, dry down, and resuspend in aqueous buffer (see Recipes) for proteomics data acquisition.

**D2. Elution (plate format)**

1. After removing the S-Trap plates, allow the plate to cool and reach room temperature before placing a collection plate spacer between the S-Trap plate and the collection plate.

2. Allow the A200 to dispense 50 μL of 50 mM ABC (or 50 mM TEAB digestion buffer). Then, apply positive pressure.

3. Add 50 μL of buffer A (see Recipes) and apply positive pressure.

4. Add 50 μL of buffer B (see Recipes), allow to stand for at least 5 min, and apply positive pressure.

5. Freeze the plate at -80 °C for at least 1 h before drying the plate using a SpeedVac concentrator at -4 °C.

6. Reconstitute when ready for LC/MS analysis using 5% acetonitrile and 0.1% formic acid.

**E. Mass spectrometry proteomics**

**Data-independent acquisition (DIA) LC-MS proteomic analysis**

Samples were separated using a Vanquish Neo UHPLC coupled to a Thermo Fisher Ascend Tribrid mass spectrometer. Samples were initially loaded onto an Acclaim PepMap C18 Trap column (7 cm total length, 2 cm bed length, 3 μm particle size, 100 A pore size) and IonOpticks Aurora Ultimate analytical column (25 cm long × 75 μm ID, 1.7 μm particle size) at a flow rate of 300 nL/min over a 60-min gradient. Solvent A consisted of LC-MS-grade water with 0.1% formic acid, and solvent B consisted of 80% acetonitrile and 0.1% formic acid. The gradient consisted of loading/starting conditions of 5% B, then to 30% B over 50 min, followed by 95% B over 5 min, finishing at 99% B for the remaining 5 min. A combined control was used for sample loading and washing. The trap column was washed using four zebra washes, and the analytical column was washed using three alternating aqueous and organic cycles before being re-equilibrated for the next sample injection. The mass spectrometry method consisted of a final spray voltage of 2,050 V and a charge state of 2. For the master scan, quadrupole isolation was selected, and Orbitrap resolution was set to 120K at a scan range of 350–1,650 m/z. A maximum injection time of 100 ms and a normalized AGC target of 50% were selected. RF lens was set to 30%, and the data type was set to profile mode. For the DIA scan, isolation utilized the quadrupole, with isolation widths of 20 m/z with a 4 m/z overlap, which led to 40 scan events. HCD fragmentation was used with a normalized CE of 28%, and the detector was set to Orbitrap at 30K resolution with a mass range of 360–1,160 m/z. Max injection time was set to 50 ms, and the AGC target was 1,000%.

## Data analysis

Spectronaut version 19.4.241104.62635; analysis type: directDIA. Process and analyze raw data using the Spectronaut^®^/DirectDIA (Biognosys) workflow with default settings. Detailed descriptions of the DirectDIA workflow have been provided in our previous publications [10,11].

By default, Spectronaut selects between local and global normalization algorithms based on the number of runs in an experiment: local normalization is applied when the number of runs does not exceed 500 (n< 500), while global normalization is used for larger experiments. In this study, the local normalization algorithm was employed. Rat UniProt FASTA database (UP000002494_10116_Rat_Reference_20240602.fasta) and Universal Contaminant Protein FASTA (created 06.01.22, used 06.03.25) were used. A false discovery rate (FDR) threshold of 1% was applied at the peptide, protein, and peptide–spectrum match (PSM) levels. Protein identifications were reported at the protein group level, and the total number of proteins was determined from protein groups passing the 1% protein-level FDR filter. This statistical FDR, together with mProphet scoring and IDPicker algorithms, minimizes potential mis-annotations. The extracted ion chromatogram (XIC) retention time window was set to dynamic, with tolerances automatically optimized by Spectronaut, and the correction factor was maintained at the system default.

Additional details regarding the DirectDIA workflow and underlying algorithms are available in the Spectronaut User Manual (https://biognosys.com/content/uploads/2023/03/Spectronaut-17_UserManual.pdf). The number of proteins identified in the 31 samples is listed in Table 1.

**Table 1.** Protein identifications in 24 rat cerebrospinal fluid samples

Sample No.	Protein identified
1	1,171
2	856
3	1,072
4	1,065
5	1,044
6	1,105
7	847
8	996
9	1,101
10	1,068
11	736
12	822
13	797
14	743
15	784
16	854
17	843
18	771
19	838
24	868
25	814
27	636
30	824
31	786

*Note: In this protocol, cerebrospinal fluid (CSF) samples were collected from rats across different groups, and the CSF volumes varied between animals, which may partially explain the variability in the number of proteins identified. In addition, samples were not normalized for total protein prior to digestion, which may further contribute to differences in protein identification. Variability may also arise from poor digestion efficiency or sample handling. To address data quality, samples were filtered based on quality control (QC) thresholds using the number of precursors (Supplemental Table 1). To highlight this, we have included a histogram of precursor identifications from each individual sample, highlighting lower identification counts in a subset of samples (Supplementary Figure 1). As these data may be reported in a separate publication, additional details are not provided here.*

## Validation of protocol

This protocol or parts of it has been used and validated in the following research articles:

- Silasi et al. [12]. Protective effects of factor XI inhibition by abelacimab in a baboon model of live Staphylococcus aureus sepsis. *Journal of Thrombosis and Haemostasis.*
- Jansen et al. [13]. Small molecule inhibition of ubiquitin C-terminal hydrolase L1 alters cell metabolism proteins and exerts anti- or pro-tumorigenic effects contingent upon chemosensitivity status in high grade serous ovarian cancer. *Frontiers in Pharmacology.*
- Biedka et al. [14]. One-pot method for preparing DNA, RNA, and protein for multiomics analysis. *Commun Biol*.
- Khadka [15]. Circulating microRNA Biomarker for Detecting Breast Cancer in High-Risk Benign Breast Tumors. *Int J Mol Sci.*
- McCoy et al. [16]. Ovarian somatic tissue rejuvenates circulating apolipoproteins and promotes cognitive health in postreproductive female mice. *GeroScience*.

## General notes and troubleshooting

**General notes**

1. Avoid multiple freeze/thaw cycles of biological samples to ensure protein integrity.

2. The peptides generated by this protocol are compatible with most liquid chromatography systems and mass spectrometry acquisition methods (e.g., DDA or DIA). DIA analysis was selected to evaluate the robustness of the S-Trap sample preparation method; however, the protocol is not limited to DIA and is equally applicable for sample preparation in DDA workflows.

3. This protocol is designed for biofluid sample preparation, including high-abundance protein depletion, digestion, and peptide cleanup. While Top 14 protein depletion improves the detection of low-abundance proteins, the protocol itself is compatible with both qualitative (identification) and quantitative proteomics analyses. The determination of protein quantification depends on the LC–MS/MS method employed.

4. Resulting peptides may also be enriched further using TiO_2_ or bead-based enrichment protocols for PTM-specific analysis.

**Troubleshooting**

**Table:** 

Problem	Cause	Solution
Incomplete depletion of high-abundance proteins (e.g., albumin, IgG)	Plasma/serum/cerebrospinal fluid sample load exceeds the binding capacity of the depletion resin.	Dilute samples or reduce the volume of protein amount of samples loaded according to the manufacturer’s instructions.
	Inadequate mixing during incubation, leading to poor resin-sample contact.	Ensure the sample and resin are mixed thoroughly at the start and maintain gentle, continuous agitation (e.g., on a tube rotator) throughout the incubation period.
	Poor sample quality leading to degradation through non-tryptic enzymatic activity.	Recollect samples. Ensure that freeze/thaw cycles are minimized and samples are immediately frozen upon collection.

## Supplementary information

The following supporting information can be downloaded here:

1. Supplementary Figure 1. Distribution of precursor identifications across 31 CSF samples.

2. Supplementary Table 1. Protein identifications and sample group information for 31 rat cerebrospinal fluid (CSF) samples
